# Does larch arabinogalactan enhance immune function? A review of mechanistic and clinical trials

**DOI:** 10.1186/s12986-016-0086-x

**Published:** 2016-04-12

**Authors:** Carine Dion, Eric Chappuis, Christophe Ripoll

**Affiliations:** Naturalpha SAS, Parc Eurasanté, 885 avenue Eugène Avinée, 59120 Loos, France

**Keywords:** Larch arabinogalactan, Common cold infections, Immune system, Vaccine, SCFA, Polysaccharides, Dietary fibers, ResistAid®, *Larix*

## Abstract

The common cold is a viral infection with important economic burdens in Western countries. The research and development of nutritional solutions to reduce the incidence and severity of colds today is a major focus of interest, and larch arabinogalactan seems to be a promising supportive agent. Arabinogalactan has been consumed by humans for thousands of years and is found in a variety of common vegetables as well as in medicinal herbs. The major commercial sources of this long, densely branched, high-molecular-weight polysaccharide are North American larch trees. The aim of this article is to review the immunomodulatory effects of larch arabinogalactan derived from *Larix laricina* and *Larix occidentalis* (North American *Larix* species) and more specifically its role in the resistance to common cold infections. In cell and animal models, larch arabinogalactan is capable of enhancing natural killer cells and macrophages as well as the secretion of pro-inflammatory cytokines. In humans a clinical study demonstrated that larch arabinogalactan increased the body’s potential to defend against common cold infection. Larch arabinogalactan decreased the incidence of cold episodes by 23 %. Improvements of serum antigen-specific IgG and IgE response to *Streptococcus pneumoniae* and tetanus vaccination suggesting a B cell dependent mechanism have been reported in vaccination studies with larch arabinogalactan, while the absence of response following influenza vaccination suggests the involvement of a T cell dependent mechanism. These observations suggest a role for larch arabinogalactan in the improvement of cold infections, although the mode of action remains to be further explored. Different hypotheses can be envisaged as larch arabinogalactan can possibly act indirectly through microbiota-dependent mechanisms and/or have a direct effect on the immune system via the gut-associated lymphoid tissue (GALT).

## Background

The common cold is an extremely common infection of the upper respiratory tract. This viral illness represents an enormous economic burden on Western society due to loss of productivity and high medical costs [1]. On average in the US, children have 6-8 and adults have 2-4 cold episodes per year [1]. Some authors estimated the economic cost of lost productivity due to the common cold as $25 billion each year ($16.6 billion due to on-the-job productivity loss, $8 billion due to absenteeism, and $230 million due to caregiver absenteeism) in the US [2]. On average each US American spends $8.30 per common cold episode on over-the-counter drugs. It is accepted that viruses, not bacteria, cause common cold infections [3] and more than 200 different types of viruses have been identified, with the rhinoviruses being the most common [4]. However, colds occasionally predispose individuals to bacterial complications. Nutrition is known to affect the immune system and can modulate resistance to infection [5]. The development of new nutritional solutions that can enhance the immune system’s response to environmental pathogens has been of major interest in recent years. Amongst these solutions, larch arabinogalactan presents the advantage of enhancing the immune function [6], and thus is speculated to protect against common colds. So far, only a few reviews have been published on arabinogalactan [6, 7], while recent studies give more insights into their effect on the immune system along with proposed mechanisms of action. The purpose of this review is to provide a comprehensive overview of the immunomodulating properties of larch arabinogalactan derived from North American *Larix* species (Eastern and Western larch) and its related mechanisms of action.

## Review

### What is arabinogalactan?

Arabinogalactans (synonyms: Galactoarabinan, Arabogalactan, Galactoarabinin) belong to a major group of carbohydrates known as hemicelluloses, which are non-starch polysaccharides that occur abundantly in the primary and secondary cell walls of plant cells and are widely spread throughout the plant kingdom.

In most plants, arabinogalactans occur in covalent association with protein, either as proteoglycans or as glycoproteins [7]. The protein moiety of arabinogalactan associated proteins is typically rich in hydroxyproline, serine, alanine, threonine, and glycine and is resistant to proteolysis in its native state, a property that is presumably conferred by extensive glycosylation [8, 9]. Arabinogalactan extracted from *Larix spp*. heartwood is an exception, as it is not bound to protein, which is evidenced by the complete absence of nitrogen during elementary analysis of *Larix laricina* [10, 11].

Arabinogalactans have been part of the human diet for thousands of years. They have been detected in seeds, leaves, roots, fruit and xylem sap of representatives of all higher plant families. Dietary sources of arabinogalactans include leek seed, carrot, radish, pear, maize, wheat and tomato [7]. Sources also include medicinal herbs such as *Echinacea* species, *Baptisia tinctoria*, *Curcuma longa*, and *Angelica acutiloba* [12] which are cultivated all over the world.

In trees, arabinogalactans are widely present as minor, water-soluble components of softwoods such as hemlock, black spruce, parana pine, mugo pine, Douglas fir, incense cedar, and juniper [13].

The major commercial sources of arabinogalactan are the North American larch trees, which are genetically different from Eurasian larch tree species [14]. The genus *Larix* (Larches) is common throughout the world. Table 1 provides an overview of the different *Larix* species that grow in specific regions [Table 1].

**Table 1:** Overview of different species of the genus *Larix* growing throughout the world

Central Europe	European larch	*Larix decidua*
Japan	Japanese larch	*Larix leptolepis/Larix kaempferi*
North America	Eastern larch, tamarack tree	*Larix laricina*
North America	Western larch	*Larix occidentalis*
Siberia	Dahurian larch/Mongolian larch	*Larix dahurica/Larix gmelinii*
Siberia	Siberian larch	*Larix sibirica*

Both the concentration and distribution of arabinogalactan varies between *Larix* species as well as within a single species, but may constitute up to 35 % by weight of dry heart wood of a larch tree [13, 15, 16]. Unique properties of larch arabinogalactan include its complete solubility and stability over a wide range of concentrations, pHs and temperatures [17].

Arabinogalactan is composed of two monomers, D-galactose and L-arabinose (in a 6:1 and 7.5:1 ratio in Western larch and Siberian larch respectively), with traces of uronic acid [7, 18]. Western larch arabinogalactan consists of a (1 → 3)-β-D-galactopyranan main chain with side (1 → 6)-linked groups of varying length to every galactosyl unit; organised as a triple helical structure with varying morphologies. These features explain why arabinogalactan forms a hydrocolloid in solution [19, 20]. The Joint FAO/WHO Expert Committee on Food Additives (JECFA) included arabinogalactan into section “Jellifying Agents, Thickening Agents, Stabilizers of Botanical Origin” and registered it under number E-409. Larch arabinogalactan was approved by the Food and Drug Administration in 1965 for direct addition to food and gained Generally Recognized As Safe (GRAS) notification in 2000. There is a Food Chemical Codex Monograph for arabinogalactan available and the larch arabinogalactan referred to here (ResistAid® brand) is produced in line with this monograph and the acceptance criteria listed therein. It is classified as a dietary fiber because it resists digestion by enzymes contained both in saliva and the small intestine, hence entering the large bowel intact, where it is fermented by the resident microflora. Larch arabinogalactan has a strong safety profile, according to a variety of toxicity studies carried out since the 1960s [10, 21].

North American larch arabinogalactan displays molecular masses ranging between 16,000 and 100,000 Daltons and presents a high molecular weight fraction (20 %), while Eurasian larch species (such as *Larix dahurica*, Mongolian larch) show neither of these characteristics [20]. In addition to composition variation existing across different species, the monosaccharide composition and molecular mass of arabinogalactan macromolecules observed can also differ within a single species depending on the specific isolation and extraction procedures employed [22]. This variability may account for the wide range of biological properties and activities documented, such as the protection of gastrointestinal mucosa and large bowel function [23], the support of digestive health by improving intestinal flora [6, 24, 25], the improvement of stress-induced gastrointestinal dysfunction [26], the effect on vascular permeability [7], the effect in metastatic disease [7] and the enhancement of immune function [7].

### Larch arabinogalactan and common cold infections: human trials

Larch arabinogalactan’s effects on the immune system have been investigated through multiple human studies with different objectives [Table 2].

**Table 2:** Summary of clinical studies on the effect of larch arabinogalactan on the immune system

Article	Extract	Challenge (Vaccine)	Subjects	Day for the measures	Parameters measured	Results: effect of the extract on parameters
Udani et al. 2013 [32]	ResistAid™ Proprietary larch arabinogalactan 1.5 or 4.5 g/day For 60 days	Tetanus & influenza vaccines Vaccination at day 30	75 healthy adults	Day 0, 45 & 60	➢ Tetanus IgG	Group 1.5 g/day, day 60: significant rise in IgG levels compared to placebo (*p* = 0.008) Group 4.5 g/day group, day 45 & 60: significant rise in IgG levels compared to baseline (*p* < 0.01) but not compared to placebo
					➢ Influenza A & B IgG & IgM	No effect
Riede et al. 2013 [30]	ResistAid™ Proprietary larch arabinogalactan 4.5 g/day For 84 days	None	199 healthy adults		Common cold episode	Reduce the incidence of common cold infection (*p* = 0.055) Reduce the number of subject affected (*p* = 0.038) Increase of the severity of symptoms (*p* = 0.028) No effect on the duration of the common cold episodes
Udani et al. 2010 [31]	ResistAid™ Proprietary larch arabinogalactan 4.5 g/day For 72 days	*Streptococcus pneumonia* Vaccination at day 30	45 healthy adults	Day 0, 51 & 72	➢ Pneumococcal IgG (subtypes 4, 6B, 9 V, 14, 18C, 19 F & 23 F)	Significant rise in IgG levels compared to placebo in 2 antibodies subtypes (18C & 23 F) at day 51 (*p* = 0.006 and *p* = 0.002) and day 72 (*p* = 0.008 and *p* = 0.041)
					➢ Pneumococcal salivary IgA	No effect
				Day 0, 30, 31, 51 & 72	➢ WBC^a^ count	No significant difference compared to placebo At day 72, rise in WBC compared to baseline (*p* = 0.045) No differences (clinically significant) in lymphocytes, neutrophils, monocytes or basophiles count Significant rise of eosinophil count at day 30 (*p* = 0.006) and day 51 (*p* = 0.014)
					➢ Inflammatory cytokines^a^: ENA-78, eotaxin, GM-CSF, IFNγ, IL10, IL12P40, IL1RA, IL2, IL4, IL5, IL6, IL8, MCP-1, MCP-3, PDGF-BB & TNF-α	Significant rise in IL6 between day 30 and 31 compared to placebo (*p* = 0.046)
					➢ Complement C3 & C4	No effect
Nantz et al. 2001 [28]	Arabinogalactan 4 g/day For 42 days	None	51 healthy adults	Day 1, 21 & 42	➢ Haematology: WBC^a^, RBC^a^, haemoglobin, hematocrit, neutrophils, lymphocytes, monocytes, platelets ➢ Count of CD19 B lymphocytes, CD4 T helper lymphocyte, CD8 T cytotoxic lymphocytes & NK1.1 natural killer cells ➢ PBMC^a^ (lymphocytes) proliferation after exposure to PMA ➢ PMN^a^ for oxidative burst activity ➢ Natural killer cells activity ➢ Ig^a^G	Significant increase of % CD8+ cells at 6 weeks after arabinogalactan compared to control group (*p* = 0.005) Significant increase in lymphocyte proliferation at 6 weeks compared to baseline in the arabinogalactan group (*p* < 0.05) only Significant effect of group detected on proportion of monocytes in the lymphocytes fraction (*p* = 0.0497), though no significant group^a^time effect detected (*p* = 0.602). No significant change in IgG levels, respiratory burst activity of neutrophils, NK cell number and B cell number.
Kim et al. 2002 [27]	Larch arabinogalactan (90 %) 1.5 g/day For 28 days	None	48 healthy female adults	Day 0 & 28	➢ Vital signs: blood pressure, radial pulse, respiration rate, temperature	No effect
					➢ Complete blood count: WBC, neutrophils, lymphocytes & monocytes	No effect
					➢ NK cells quantitative	No effect
					➢ Complement properdin	No effect
					➢ TNF-α	Significant decrease (*p* = 0.044)
					➢ EBV VCA IgG Ab ➢ CMV IgG Ab	No effect No effect
					➢ *Lactobacillus acidophilus* stool culture ➢ Stool fungus culture for yeast	No effect No effect
					➢ Health related quality of life (SF-36)	Increase of the bowel movement (75 % of the subjects affected)
Kim et al. 2002 [12]	Larch arabinogalactan (90 %) – different concentration grades 1.5 & 4.5 g/day For 28 days	None	21 healthy adults	Day 0 & 28	➢ Haematology: WBC^a^, RBC^a^, haemoglobin, hematocrit, monocytes	No effect
					➢ TNF-α ➢ IFN-γ ➢ IL6	No effect No effect No effect
					➢ Stool culture	No effect
					➢ Health related quality of life (SF-36)	No effect

^a^ *ENA* epithelial neutrophil activating peptide-78, *GM-CSF* granulocyte monocytes colony stimulating factor, *IFNγ* interferon gamma, *IL* interleukin, *MCP* monocyte chemotactic protein-1, MCP-3, *PDGF* platelet-derived growth factor-BB or *TNF* tumour necrosis factor-alpha, *PBMC* peripheral blood mononuclear cells, *PMN* polymorphonuclear neutrophils, *PMA* phorbol 12-myristate 13-acetate, *WBC* White blood cells, *RBC* red blood cells, *Ig* immunoglobulin, *EBV VCA IgG Ab* Epstein-Barr Virus viral capsid antigen IgG antibody, *CMV IgG Ab* Cytomegalovirus IgG antibody, *Ab* antibody

Three clinical trials performed in free-living healthy adults were retrieved from the literature. Two of these studies explored the effect of larch arabinogalactan on TNF-α in serum following four weeks’ supplementation at 1.5 g/d. Results from both studies displayed different results as one reported an increase on this parameter while the other did not. Furthermore, other immune parameters explored (NK cells, immunoglobulins, immune cells counts) were not affected by the supplementation in either trial [12, 27]. The third study performed with a higher dose of larch arabinogalactan (4 g/d) in 51 young healthy adults did not evaluate the previous parameters but rather demonstrated that 4 g/d of larch arabinogalactan provided for 6 weeks in orange juice significantly increased the percentage of blood CD8^+^ T-suppressor cells compared to a placebo (*p* = 0.005) and increased the proportion of monocytes in the lymphocyte fraction (*p* = 0.05), independent of time. Lymphocyte proliferation was significantly increased at 6 weeks compared to baseline in the arabinogalactan group, which was not the case in the control group. Other parameters including serum IgG levels, respiratory burst activity of neutrophils, NK cell number and B cell number remained unchanged [28]. These three studies performed in healthy adults suggest that larch arabinogalactan might influence TNF-α secretion and modulate the proportion of immune cells proportions while other parameters such as immunoglobulin levels, NK cells levels and activity or neutrophils activity seemed unaffected by the supplementation, though the pattern of effects exerted was different between studies. In these clinical trials however, the relevance of the model (healthy subjects and absence of immune challenge) and markers could be questioned, as improvement of immune response can be observed mainly in immune-challenged conditions. As discussed in an expert’s review, the markers providing the most useful indication to assess the immune-modulating properties of nutraceuticals are those that involve either a standard assessment of relevant symptoms (symptoms of allergies or common infections) or those involving in vivo responses to a defined challenge with allergens or antigens (allergen provocation, vaccine response) [29].

Larch arabinogalactan has been tested in several of these immune-challenge models. Riede et al. evaluated the effect of larch arabinoglalactan on common cold infections in healthy adults. This placebo-controlled, double-blind and randomised trial was performed during the cold season of 2010/2011 with 199 healthy volunteers who had reported at least 3 upper respiratory tract infections in the last 6 months. After daily administration of either 4.5 g of an arabinogalactan preparation or placebo over a period of 12 weeks, it appeared that larch arabinogalactan (ResistAid® brand) increased the body’s potential to defend against infections [30]. The incidence of common cold infections in the group supplemented with arabinogalactan was significantly decreased compared to the placebo group in both analysis sets: full analysis set (FAS, *p* = 0.038) and Per Protocol (PP, *p* = 0.033). The number of cold episodes strongly tended to decrease in the arabinogalactan group in the FAS (*p* = 0.055), while in the PP analysis this decrease of 23 % was statistically significant (*p* = 0.04) [Table 3] [30]. A trend for a reduction in the duration of cold episodes was observed in supplemented subjects (*p* = 0.061). Interestingly, self-reported severity of cold symptoms was higher on the first day of cold episodes in subjects supplemented with arabinogalactan while this difference was not observed on the fifth day of cold episodes [30]. It has been suggested that the highly variable subjective perception of a disease could be responsible for the difference noticed. However, these results could also be explained by a quicker and stronger immune response favoured by the supplementation with arabinogalactan. Therefore, the common symptoms of a cold such as redness, heat, swelling, and pain, experienced more intensely by participants on the first day of the trial could be attributed to such an immune response.

**Table 3:** Summary of Riede et al.’s results on the effect of larch arabinogalactan on common cold

Population analysed	FAS^a^		PP^a^ set	
Groups	Placebo	AG^a^	Placebo	AG^a^
Number of common cold episodes	1.06 ± 0.85	0.83 ± 0.82	1.10 ± 0.85	0.85 ± 0.82 *
Number of subjects affected by a cold episode	72.4 %	58.4 % *	74.4 %	59.8 % *
Duration of common cold episodes	8.3 ± 2.9	8.5 ± 2.8	-	-
Intensity of symptoms after 5 days, documented in CRF^a^	8.5 ± 6.6	8.4 ± 6.8	-	-
Intensity of symptoms after 5 days, from subject diary	5.85 ± 8.35	4.73 ± 8.08	-	-
Intensity of symptoms at start, documented in CRF^a^	11.6 ± 6.3	13.3 ± 6.6	-	-
Intensity of symptoms at start, from subject diary	11.5 ± 6.5	13.7 ± 6.9 *	-	-

^a^ *AG* Arabinogalactan, *CRF* Case Report Form, *FAS* Full analysis set, *PP* Per protocol

Mean values (± SD) significantly different from the placebo: * *p* < 0.05

More specific information on the enhancement of an immune response following a challenge has been obtained using the vaccine challenge method. The impact of a 10-week supplementation period with 4.5 g/d of a proprietary arabinogalactan preparation from larch tree (ResistAid® brand) was studied in a vaccine model [31]. The researchers demonstrated that the preparation selectively enhanced the antibody response to vaccination against *Streptococcus pneumoniae* and observed an increase in pneumococcal IgG antibodies of various pneumococcal antigens [31].

A similar study performed by the same research group compared the effectiveness of the ResistAid® ingredient at a daily dose of 1.5 g to a placebo, and demonstrated a significant increase in IgG antibody response to tetanus vaccine, while no improvement was observed following influenza vaccine [32].

These results taken together suggest that larch arabinogalactan can improve immunity by decreasing infections and improving immunoglobulin response following a standardized immune challenge. Doses used in these trials suggest that larch arabinogalactan may improve immune response at a dose as low as 1.5 g/d taken for several weeks; however, more consistent results have been obtained at a dose level of 4.5 g/d over several weeks. This was seen both on vaccine models and on infection-prevention models. Further clinical studies would be required in order to confirm and clarify these findings, such as the lack of response following influenza vaccine.

### Effect of larch arabinogalactan on immune parameters: preclinical studies

The immunostimulatory activity of larch arabinogalactan has been investigated in various in vitro and in vivo studies. These works have demonstrated activation of different components of the immune system. An effect on natural killer cells (NK cells), components of the nonspecific immediate immune response to antigens, has been observed. Hauer and Anderer’s ex vivo study, using human peripheral blood mononuclear cells (PBMC), demonstrated larch arabinogalactan’s ability to enhance NK cells’activity/cytotoxicity (i.e. ability to mediate spontaneous cytotoxicity against tumour cells and virus-infected cells without prior sensitisation by antigen) through a possible increase in interferon-gamma (IFN-γ) [33]. The investigators also highlighted larch arabinogalactan’s ability to induce the production and/or release of pro-inflammatory cytokines such as tumour necrosis factors-alpha (TNF-α), Interleukin-1 beta (IL-1β) and Interleukin-6 (IL-6) [33]. It has been shown that tumoricidal and phagocytic activities of macrophages are enhanced by treatment with larch arabinogalactan, and these activated cells exhibit increased production of nitric oxide (NO), H_2_O_2_, TNF-α and IL-6 [34]. Furthermore, some but not all arabinogalactan-containing polysaccharides from other sources have been shown to have complement-fixing activity contributing to their immune-modulating effects [35].

Studies done in vivo report that the number of mouse spleen NK cells more than double compared to control after 14 days exposure to intra-peritoneally injected larch arabinogalactan [36]. The role played by larch arabinogalactan on the innate immune system is further substantiated by Grieshop et al.’s in vivo study on dogs, demonstrating that oral administration of larch arabinogalactan (at doses of 0.55 g/day or 1.65 g/day for 10 days) increases the number of circulating white blood cell counts, namely neutrophils and eosinophils [17]. The effect of larch arabinogalactan on the adaptive immune system has also been studied. Grieshop et al.’s study on dogs showed that the number of lymphocytes (CD4^+^T helper, CD8^+^ cytotoxic T cells or B CD19^+^) was not affected by larch arabinogalactan administration. Serum IgG, IgM and IgA were also unaffected [17]. However, Choi’s group affirms that the treatment of mice splenic lymphocytes with arabinogalactan increased their cytotoxic activity against tumour cells [34].

### Pharmacokinetics of larch arabinogalactan

A number of studies aimed to investigate whether and how arabinogalactan reaches the systemic circulation in order to exert its effects on immunity. Larch arabinogalactan is resistant to digestion by enzymes in the upper gastrointestinal tract. It reaches the colon where it is slowly fermented by the gastrointestinal microflora and thus, promotes the growth of indigenous intestinal microflora such as *Bifidobacterium* and *Lactobacillus acidophilus* [12, 17, 25, 37] similarly to other oligosaccharides [38]. The fermentation of acacia gum arabinogalactan occurs both in proximal and distal parts of the colon while other oligosaccharides such as fructooligosaccharides may be fermented mostly in the proximal part of the colon as shown with an in vitro model of the human intestinal microbial ecosystem [39].

**Fig. 1:**
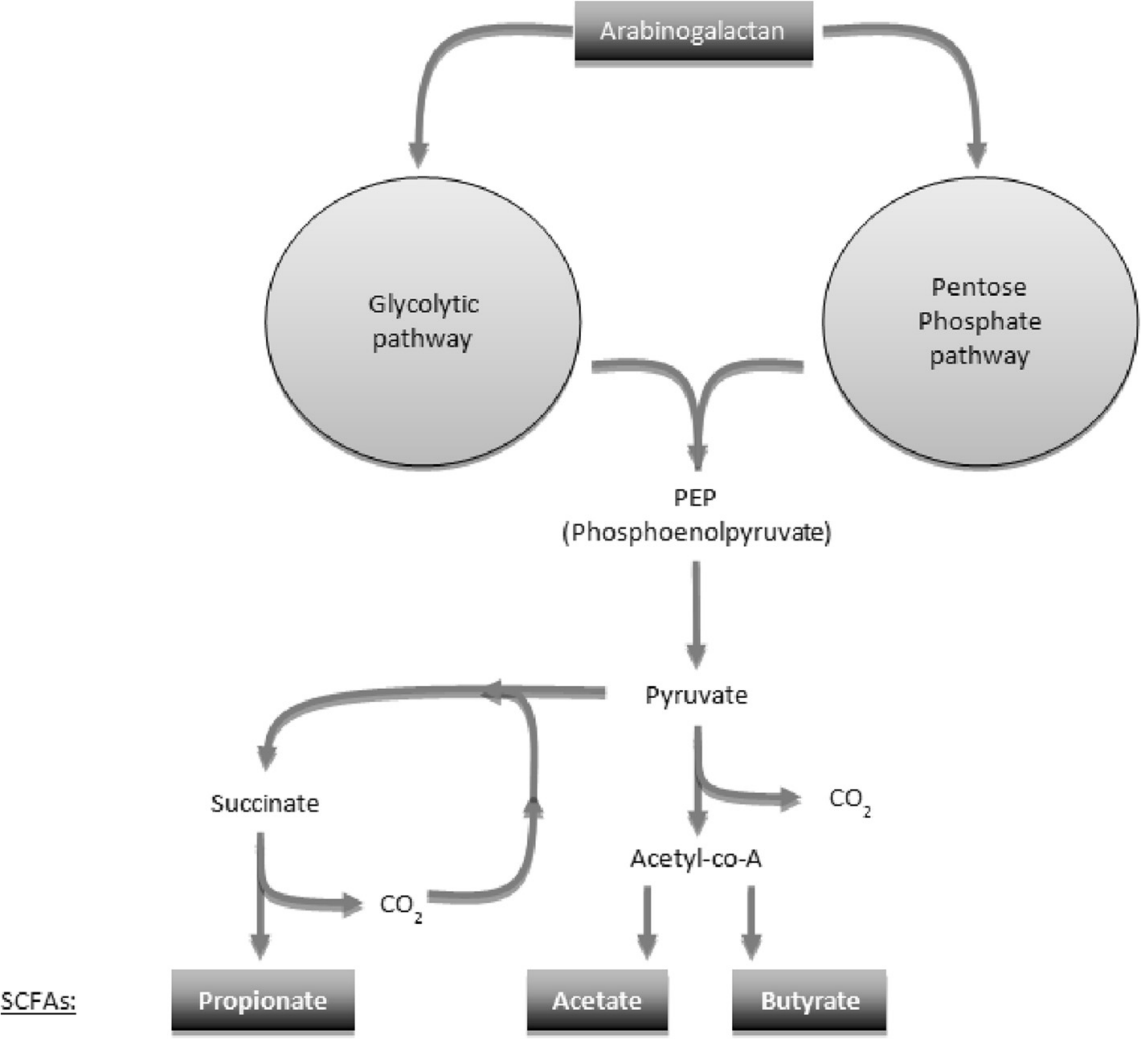
Proposed mechanisms of action of larch arabinogalactan on immune system

Carbohydrates of plant fibers are known to be digested to varying degrees by the large bowel flora [40] and *Isphagula* husk (an arabinoxylan of similar structure to arabinogalactan) metabolization by the gut flora reaches 85-100 % in humans [24, 41]. Moreover, Vince et al. have used an in vitro faecal incubation system and suggest complete fermentation of acacia gum arabinogalactan after 48 h [24]. The fermentation by the resident colonic microflora of larch arabinogalactan results in the production of the short chain fatty acids (SCFA) [Fig. 1], butyrate, acetate and propionate [12, 17, 24], with the latter two being predominantly produced [42]. Apart from this pathway, the existence of a transfer of the whole molecule of arabinogalactan to the systemic immune system via the M-cells of the GALT [34] is supported by the study of Yamashita et al. [43] on antitumor peptidomannan KS-2, providing evidence that orally administrated polysaccharides could be absorbed via portal vein and intestinal lymphatics into the general circulation with an intact molecular size.

According to these elements of evidence, arabinogalactan may potentially exert its effects indirectly, relying on SCFAs actions, or directly as a circulating agent.

### Potential modes of action involved

**Fig. 2:**
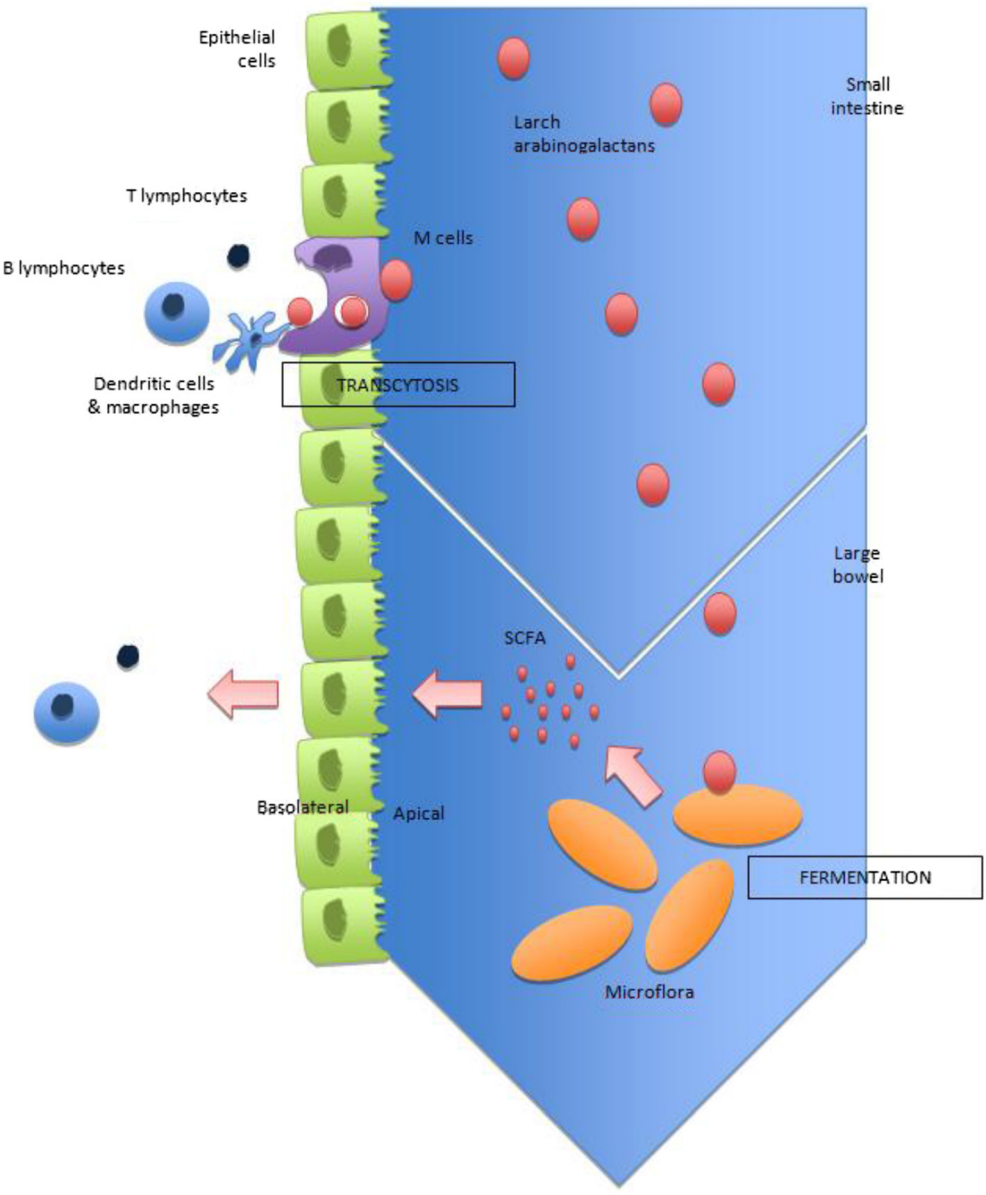
Larch arabinogalactan metabolism: simplified diagram of polysaccharide breakdown and the main routes of carbohydrate fermentation in the large intestine

Studies of the specific modes of action of larch arabinogalactan support in part the two pathways developed above. Indeed, arabinogalactan (similarly to other gut-fermented polysaccharides) can possibly act indirectly through microbiota-dependent mechanisms (i.e. rebalancing microbiota composition in the gut, production of SCFAs) and/or have a direct effect on the immune system after passage from the gut lumen through the GALT [Fig. 2].

The gut fermentation pathway generates SCFAs at high concentrations through the breakdown of carbohydrates [44]. These compounds, rapidly absorbed from the colonic lumen, enter the portal and peripheral circulation [45], regulate the metabolism, proliferation and differentiation of colonic epithelial cells [46] as well as intestinal immunity [38]. Their interactions with G-protein-coupled receptors 41 and 43 (GPR41 and 43), expressed on a range of immune cells [47, 48] may affect inflammatory responses [48]. SCFAs regulate the leukocyte production of cytokines, such as TNF-α, IFN-γ, IL-2, IL-6 and IL-10, as well as eicosanoids and chemokines (e.g., MCP-1 and CINC-2) [49, 50] and butyrate also affects leukocyte chemotaxis, limiting the migration and, thus, the microbial pathogens’ destruction [49]. However, their exact and individual role in these effects remains unclear. This particularly applies to propionate and acetate, which are the two SCFAs predominantly generated by arabinogalactan fermentation [45]. In addition, Choi et al. suggested that mono- and disaccharides generated from complex carbohydrates during digestion could also exert an immunostimulating role, despite little evidence supporting the influence of simple carbohydrates on immune parameters [34].

There is also a possibility that larch arabinogalactan expresses its clinical effects as intact macromolecules rather than as fragments resulting from digestion [34], though the mode of action involved is still unclear. According to this second possible mode of action, complex carbohydrates could exert an effect on gut-associated immunity in the small intestine. This part of the gut contains the GALT, consisting of immunoreactive cells and organized lymphoid tissues, found in close contact with the mucosal lining of the gut, and thus the lumen. M-cells are specialised epithelial cells found in the follicle-associated epithelium (FAE) and continuously sample the lumen of the small intestine [51]. Soluble proteins, particles and live microorganisms traverse the M-cells by transcytosis and are delivered into a “pocket” on the basolateral side of the cell that is packed with T and B lymphocytes, macrophages and dendritic cells [51, 52]. Antigens seem to be unaltered by this translocation [52] and once across the M-cell, it is processed by antigen-presenting cells (APC) and presented to T lymphocytes that proliferate in place and stimulate local B lymphocytes [52]. These then migrate to distant sites [52], thus playing an important immunomodulatory role.

Despite these proposed mechanisms of action, several findings from clinical studies remain to be explained. As evidenced by Udani’s research group, arabinogalactan supplementation failed to enhance immune response following influenza vaccine, suggesting that this polysaccharide confers a benefit in preparing the immune system to manage infection with bacterial antigens, but perhaps not with viral antigens [32]. Udani hypothesizes that arabinogalactan is capable of stimulating the Peyer’s patches in the gut as it traverses the intestines. The larch polysaccharides may have a similar structure to these potentially pathogenic bacteria, and therefore, provide a low level of stimulation, which keeps an array of antibodies ready in case the actual antigen appears [32]. This hypothesis is consistent for the *Streptococcus pneumoniae* vaccine, as the vaccine is made of bacterial sugars from 23 pneumococcal types. Vaccines produced from bacterial polysaccharides are generally known to trigger T-independent responses, i.e., directly inducing a B cell response in the absence of T cell help. Other features of this response include absence of immune memory and induction of low-affinity antibodies [53]. However, the effect of larch arabinogalactan on tetanus vaccine response seems to be due to other mechanisms that need to be identified. The tetanus vaccine is composed of toxoids, a modified and harmless form of the tetanus toxin protein (also named tetanospasmin and produced by *Clostridium tetani*). The protection is often mediated by B lymphocytes and IgG, as observed for *Streptococcus pneumoniae* and tetanus vaccination [53]. However, T cells could also be an important or the main effector of the immune response, as it is the case for tuberculosis vaccine (CD4^+^ T cells) or live attenuated influenza intranasal vaccine (CD8^+^ T cells) [53]. Thus, it is possible that arabinogalactan acts differently on these various immune cell types, influencing the efficiency of the vaccination through many different mechanisms. The latter assertion is consistent with the effects exerted by other plant polysaccharides that present the capacity to positively modulate the influenza vaccine response. A series of studies performed by Vos et al. shows that a mixture of oligosaccharides, consisting of short-chain galactooligosaccharides (scGOS) and long-chain fructooligosaccharides (lcFOS), influenced immune response to an influenza vaccine in mice by increasing vaccine-specific delayed-type hypersensitivity (DTH) response and modulating the lymphocyte T helper Th1/Th2 balance through enhancement of Th1-related and suppression of Th2-related parameters [54–57]. Regarding influenza vaccination, the hypothesis that the main immune cell type involved is T cells is supported by the results obtained in Bunout et al.’s clinical study, showing no influence of fructooligosaccharide consumption by healthy elderly on immunoglobulin levels (IgA, IgM, IgG and salivary secretory IgA) after influenza vaccine [58], which is consistent with Udani’s results on arabinogalactan [32]. To date, a beneficial immunological effect of larch arabinogalactan was shown following challenges with *Streptococcus pneumoniae* and tetanus vaccination only, through increased concentration of antigen-specific IgG and IgE antibodies in serum. In future investigations, the study of different antibody isotypes could provide additional information on the type of immune response elicited (IgG1 and IgG3 indicating Th1-driven responses and IgG4 and IgE indicating Th2-driven responses) [5]. While measuring serum immune markers reflects in vivo response [5], measuring antibody production would allow to investigate the effect of larch arabinogalactan at the functional level. Regarding influenza, it is not obvious to identify a role for arabinogalactan in the improvement of the vaccine effect using serum immunoglobulins as biomarkers. However, the study of markers such as lymphocyte activation (i.e. surface expression of activation markers on CD8^+^ lymphocytes) or lymphocyte-derived mediators (i.e. production of cytokines) could be more appropriate according to the mode of action involved.

## Conclusion

Common cold infections are both a health problem and economic problem in Western countries, hence, it is important to develop supportive solutions. Recent human studies have demonstrated that dietary intervention with arabinogalactan from North American *Larix* species could increase resistance to infections. Larch arabinogalactan seems to positively influence NK cells, macrophage activities and pro-inflammatory cytokine production. A clinical study demonstrated that larch arabinogalactan supplementation reduced the incidence of common cold infections. In two vaccine models (*Streptococcus pneumoniae* and tetanus), larch arabinogalactan had an immunostimulatory effect. Therefore, these results suggest a role for larch arabinogalactan in the improvement of immune system and defence against pathogens in humans. It is interesting to note that both models (infection and vaccine) are considered relevant by the European Food Safety Authority (EFSA) to substantiate health claims on immune system in the frame of European regulation (EC) 1924/2006 on nutrition and health claims [59, 60].

To explain the mode of action, it has been suggested that it can interact with the immune system either indirectly through the production of SCFAs that affect inflammatory responses via leukocytes function and cytokine production, or directly through the capacity of M-cells to transfer intact arabinogalactan through the intestinal barrier, delivering it to immune cells (APC). However, the exact mode of action is not yet completely understood and further studies are required to better understand it and define more precisely the benefits of larch arabinogalactan to the immune system.

## Abbreviations

APC: antigen-presenting cells
CD: cluster of differentiation
CINC-2: cytokine induce neutrophil chemoattractant-2
DTH: delayed-type hypersensitivity
EFSA: European food safety authority
FAE: follicle-associated epithelium
FAS: full analysis set
GALT: gut-associated lymphoid tissue
GPR: G-protein-coupled receptors
GRAS: generally recognized as safe
i.p.: intraperitoneal
IFN-γ: interferon-gamma
Ig: immunoglobulin
IL: interleukin
lcFOS: long-chain fructooligosaccharides
MCP-1: macrophage chemoattractant protein
NK cells: natural killer cells
NO: nitric oxide
pAOS: pectin derived acidic oligosaccharides
PBMC: peripheral blood mononuclear cells
PP: per protocol
SCFA: short-chain fatty acids
scGOS: short chain galactooligosaccharides
Th: T helper
TNF-α: tumour necrosis factors-alpha
Tregs: regulatory T-cells.
